# Characterization of the Interaction between Human α_1_‑Acid Glycoprotein and Apremilast Enantiomers: HPLC, Isothermal Titration Calorimetry, Ultracentrifugation, and Docking Studies

**DOI:** 10.1021/acsomega.5c07756

**Published:** 2025-12-09

**Authors:** Gergely Dombi, Béla Fiser, Adam Buczkowski, Artur Stepniak, Levente Tyukodi, Zsuzsanna Rozmer, András Lukács, Zoltán-István Szabó, Miklós Poór, Gergő Tóth

**Affiliations:** † Department of Pharmaceutical Chemistry, 37637 Semmelweis University, Hőgyes E. u. 9, 1092 Budapest, Hungary; ‡ Center for Pharmacology and Drug Research & Development, 37637 Semmelweis University, Üllői u. 26, 1085 Budapest, Hungary; § Institute of Chemistry, 61764 University of Miskolc, Egyetem u. 1, 3515 Miskolc, Hungary; ∥ Department of Biology and Chemistry, Ferenc Rákóczi II Transcarpathian Hungarian College of Higher Education, Berehove 6, Transcarpathia, 90201 Beregszász, Ukraine; ⊥ Department of Physical Chemistry, Faculty of Chemistry, 49602 University of Lodz, Pomorska 163/165, 90-149 Łódź, Poland; # Department of Pharmaceutical Chemistry, 37656 University of Pécs, Rókus u. 2, 7624 Pécs, Hungary; ∇ Department of Biophysics, Medical School, 37656 University of Pécs, Szigeti út 12, 7624 Pécs, Hungary; ○ “George Emil Palade” University of Medicine, Pharmacy, Science, and Technology of Târgu Mureş, Strada Gheorghe Marinescu 38, 540139 Târgu Mureş, Romania; ◆ Sz-imfidum Ltd, Lunga nr. 504, 525401 Lunga, Romania; ¶ Department of Laboratory Medicine, Medical School, 37656 University of Pécs, Ifjúság útja 13, 7624 Pécs, Hungary; ⬡ Molecular Medicine Research Group, János Szentágothai Research Centre, 37656 University of Pécs, Ifjúság útja 20, 7624 Pécs, Hungary

## Abstract

The interactions between human α_1_-acid glycoprotein (AGP) and the enantiomers of apremilast (APR) were investigated by using a combination of HPLC, isothermal titration calorimetry (ITC), ultracentrifugation, and molecular docking. HPLC analyses on a chiral AGP column revealed enantioselective binding, with S-APR showing slightly higher stability for AGP compared with R-APR. However, the stability difference is sufficient for the baseline separation of APR enantiomers. ITC experiments confirmed the spontaneous and exothermic nature of the binding, with similarly high binding constants (log K > 5) for both enantiomers but a more exothermic enthalpy for the S-form. Ultracentrifugation studies further supported the high stability of the AGP–APR complexes, with the S-enantiomer again demonstrating marginally stronger binding. Molecular docking revealed that S-APR formed more direct interactions, including additional hydrogen bonds and π–π interactions with AGP compared to R-APR, corroborating the experimental findings. This enhanced interaction also contributes to the chiral separation. Comparison of the protein binding data suggests that AGP plays a significant role in APR transport. Altogether, the multianalytical approach provided detailed insight into the enantioselective binding of APR to AGP, which may contribute to understanding its pharmacokinetic behavior and therapeutic action.

## Graphical abstract

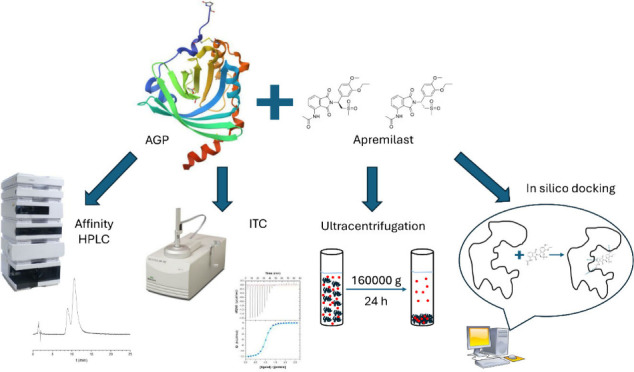

## Introduction

Plasma protein binding plays a critical role in the pharmacokinetics, distribution, therapeutic efficacy, and bioavailability of drug compounds. Therefore, investigating the binding interactions between drugs and plasma proteins is essential for a better understanding of drug transport processes *in vivo*.^1,2^ Among plasma proteins, serum albumin is the most abundant in human blood and primarily binds acidic and neutral drugs.^3^ In contrast, human α_1_-acid glycoprotein (AGP) is an acute-phase serum protein with broad biological functions, including immunomodulation and drug binding.^4^ Although AGP is present at concentrations approximately 30 times lower than that of albumin, it is a key plasma protein involved in the binding and transport of basic and some neutral drugs. It should also be noted that the concentration of AGP in blood increases up to 5 times during inflammation or malignancies.^5,6^ Numerous studies have also demonstrated the important role of AGP in drug binding *in vitro*. Structurally, AGP consists of a single polypeptide chain composed of 183 amino acids and five *N*-linked glycans attached to asparagine residues. AGP isolated from plasma exhibits structural heterogeneity, primarily due to extensive glycosylation and genetic polymorphism. Most individuals express a combination of two or three major genetic variants—F1, S, and/or A—encoded by two distinct genes, leading to a complex mixture of AGP isoforms in systemic circulation. Commercially pooled AGP can be fractionated through chromatography, yielding approximately 70% of an F1 and S mixture (F1*S) and 30% of variant A. It has also been demonstrated that the ligand binding characteristics of these variants are different.^7^

Although most studies in the literature have traditionally focused on drug binding to human serum albumin (HSA), a growing number of investigations over the past decade have examined the interactions between AGP and various pharmaceutical compounds.^8–12^ There are many methods for investigating protein ligand interactions; however, it should be noted that a complete picture can only be achieved by using multiple techniques, as each method has its own advantages and disadvantages.^13^ High-performance affinity chromatography is widely used to study protein–ligand interactions in drug binding. The target protein is immobilized on a solid support to form the stationary phase; analytes with stronger affinity are retained longer, enabling the estimation of binding from retention factors.^14–16^ However, immobilization can partially denature proteins or alter their conformation, and active-site orientation and accessibility may vary with the coupling chemistry, reducing physiological relevance and reproducibility. Thus, high-performance affinity chromatography data should be interpreted cautiously and, when possible, corroborated with orthogonal methods that do not require immobilization.^15,17^ Complementary techniques could include ultrafiltration, ultracentrifugation, and calorimetric methods including isothermal titration calorimetry (ITC). Among them, ITC is especially valuable for its ability to directly determine binding constants and thermodynamic parameters, although it requires excessive amounts of protein and ligand.^18,19^ Analytical ultracentrifugation separates species by sedimentation under high centrifugal fields without disrupting ligand–protein complexes and has been applied to HSA and other proteins, including AGP.^20–23^ Ultracentrifugation also avoids membranes, preventing ligand adsorption common in ultrafiltration or equilibrium dialysis, and can accommodate multiple ligands simultaneously.^22,24^ Spectroscopic methods (e.g., fluorescence quenching) are sensitive but can be confounded by high native fluorescence.^25,26^ NMR and X-ray provide atomic-level insights yet are limited by protein size and crystallization requirements, respectively.^9,10,27,28^ Complementarily, computational modeling enables visualization and prediction of drug–protein interactions at the molecular level.^29,30^ Characterizing protein–ligand interactions requires the integration of diverse analytical techniques, each contributing unique strengths to ensure reliable and validated results. For instance, the chromatographic method is well-suited for rapid enantioselective binding studies; ultracentrifugation and calorimetry can be used for the accurate determination of stability constants and thermodynamic characterization of binding, while *in silico* modeling elucidates the binding process at the molecular level.

Apremilast (APR) is an innovative, FDA-approved derivative of thalidomide that acts as a selective inhibitor of phosphodiesterase-4 (PDE4).^31^ It is utilized in the management of inflammatory diseases including psoriasis and psoriatic arthritis. As a PDE4 inhibitor, APR prevents the degradation of cyclic adenosine 3′,5′-monophosphate (cAMP). The resulting increase in intracellular cAMP levels in PDE4-expressing cells modulates the immune response by downregulating pro-inflammatory mediators (e.g., TNF-α and IL-23) and upregulating anti-inflammatory cytokines such as IL-10.^32^ Structurally related to thalidomide, containing the same phthalimide moiety, APR contains a single asymmetric carbon atom, giving rise to two enantiomers. In contrast to thalidomide, APR does not possess an acidic hydrogen at its asymmetric center, diminishing its tendency to undergo racemization under physiological conditions. The structure of APR enantiomers is depicted in Figure 1. The more effective S-APR is marketed.

**Figure 1:**
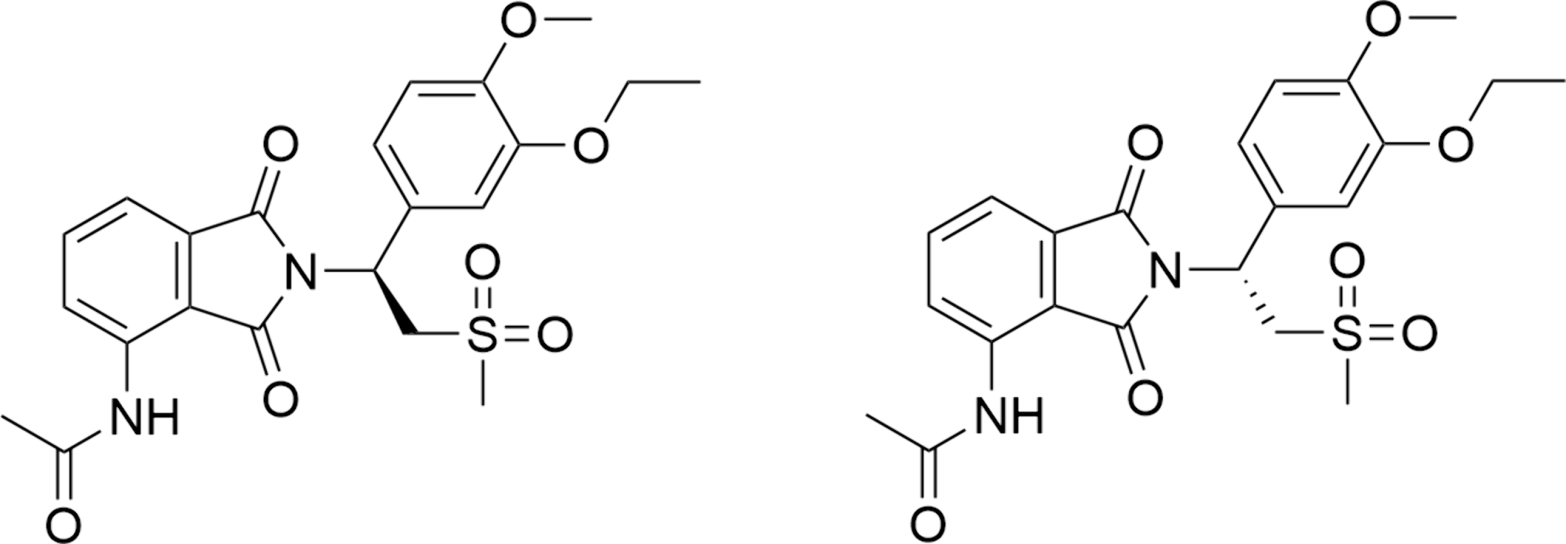
Chemical structures of apremilast (APR) enantiomers. Left: the more potent S-APR (eutomer); right: R-APR (distomer).

In our recent work, the enantioselective binding of APR to HSA was characterized.^33^ However, with APR as a neutral molecule, it is possible that it can bind to AGP. As a continuation of our recent work, the objective of this study was to characterize the AGP–APR complexes using HPLC, ultracentrifugation, ITC, and molecular modeling with special attention to the enantioselective processes. A comparative analysis was also performed on the binding of the drug molecule toward both AGP and HSA.

## Results and Discussion

### Enantioselective Binding by HPLC

HPLC employing a protein-based chiral stationary phase (CSP) offers a streamlined and rapid method for assessing the binding of compounds to immobilized proteins. Enantioseparation occurs due to differences in the binding of enantiomers to the CSP, enabling the characterization of enantioselective interactions, even from racemic mixtures.^34,35^

In this study, AGP was used as the chiral selector. Binding was evaluated under isocratic conditions using sodium phosphate, ammonium carbonate, and ammonium acetate buffers (pH 7.0 at 10 mM concentration) as the aqueous component of the mobile phase with 2-propanol serving as the organic modifier. Representative chromatograms are shown in Figure 2, with a summary of the results provided in Table 1.

**Figure 2:**
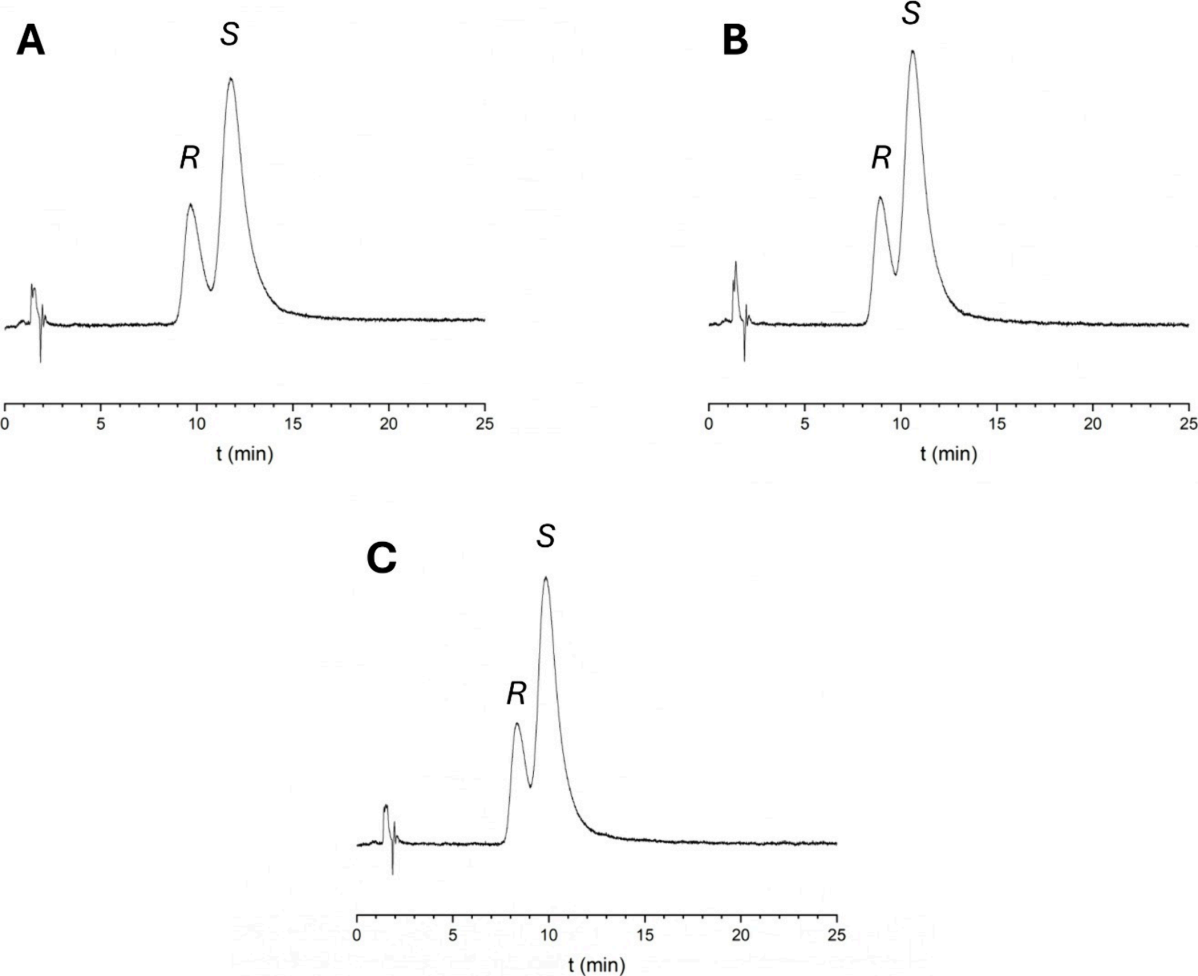
Some representative chromatograms from the HPLC study on a chiral AGP column at 25 °C. R and S denote the enantiomers of APR. (A) Eluent is 95% sodium phosphate buffer 5% IPA. (B) Eluent is 95% ammonium acetate buffer 5% IPA. (C) Eluent is 95% ammonium bicarbonate 5% IPA. Flow rate: 0.7 mL/min. The detection wavelength was set at 230 nm.

**Table 1:** Results of Chromatographic Measurements for Different Buffer Types^a^

Buffer type	Buffer ratio in eluent (%)	k_R_	k_S_
Sodium phosphate	95	3.99 ± 0.02	5.05 ± 0.03
	92.5	1.69 ± 0.01	2.00 ± 0.01
	90	0.98 ± 0.01	0.98 ± 0.01
	85	0.34 ± 0.01	0.34 ± 0.01
Ammonium acetate	95	3.29 ± 0.02	4.07 ± 0.02
	92.5	1.51 ± 0.03	1.72 ± 0.03
	90	0.90 ± 0.01	0.90 ± 0.01
	85	0.33 ± 0.01	0.33 ± 0.01
Ammonium bicarbonate	95	3.59 ± 0.02	4.44 ± 0.02
	92.5	1.62 ± 0.01	1.89 ± 0.01
	90	0.94 ± 0.01	0.94 ± 0.01
	85	0.32 ± 0.01	0.32 ± 0.01

a k_R_ is the retention factor of the R-enantiomer, while k_S_ is the retention factor of the S-enantiomer.

The enantiomers of APR were successfully separated on the AGP column under all three buffer conditions, indicating enantioselective binding. However, increasing the proportion of the organic modifier led to reduced resolution, suggesting that the difference in the binding strength between the enantiomers was modest. Based on the elution order, the S-enantiomer of APR showed higher stability for AGP than its R-counterpart.

HPLC columns with immobilized proteins can also be used to estimate the percentage of the drug bound to the protein. A plot of the natural logarithm of the retention factor (ln k) versus the volume fraction of the organic modifier revealed a logarithmic trend. Extrapolating this relationship to a zero organic modifier approximates physiological conditions. The intercept of the regression lines was used to calculate protein binding percentages based on eq 2.

The calculated protein binding exceeded 98% for both enantiomers, with the difference in binding percentages between them being approximately 1%. The binding percentage values for the different eluents used are listed in Table 2. Nevertheless, enantioseparation was achieved, confirming detectable differences in binding interactions. The type of buffer had a minimal impact on the binding percentages, as comparable values were observed under all tested conditions. The lowest binding was recorded in ammonium acetate, while the highest was observed in sodium phosphate.

**Table 2:** Bound Percentages (b%) of APR Enantiomers Calculated with Different Buffers

Buffer type	b%_R‑APR_	b%_S‑APR_
Sodium phosphate	99.31	99.63
Ammonium bicarbonate	99.19	99.54
Ammonium acetate	98.37	99.01

### Stoichiometry and Binding Constant

#### ITC Study

The thermal effects of titration of AGP protein (10 μM) with the studied ligands (500 μM S-APR or R-APR) were studied in aqueous 5% DMSO solution at 25 °C, using ITC (Figure 3A,B). The corresponding effects of S-APR (or R-APR) dilution in the solvent were determined independently and subtracted from the heat effects of AGP with S-APR (or R-APR) titration. Obtained heat effects of direct interactions of AGP with S-APR and R-APR ligand were described (Origin MicroCal) with multiparameter nonlinear regression (Figure 3C) using the One Set of Sites model. The determined parameters are summarized in Table 3.

**Figure 3:**
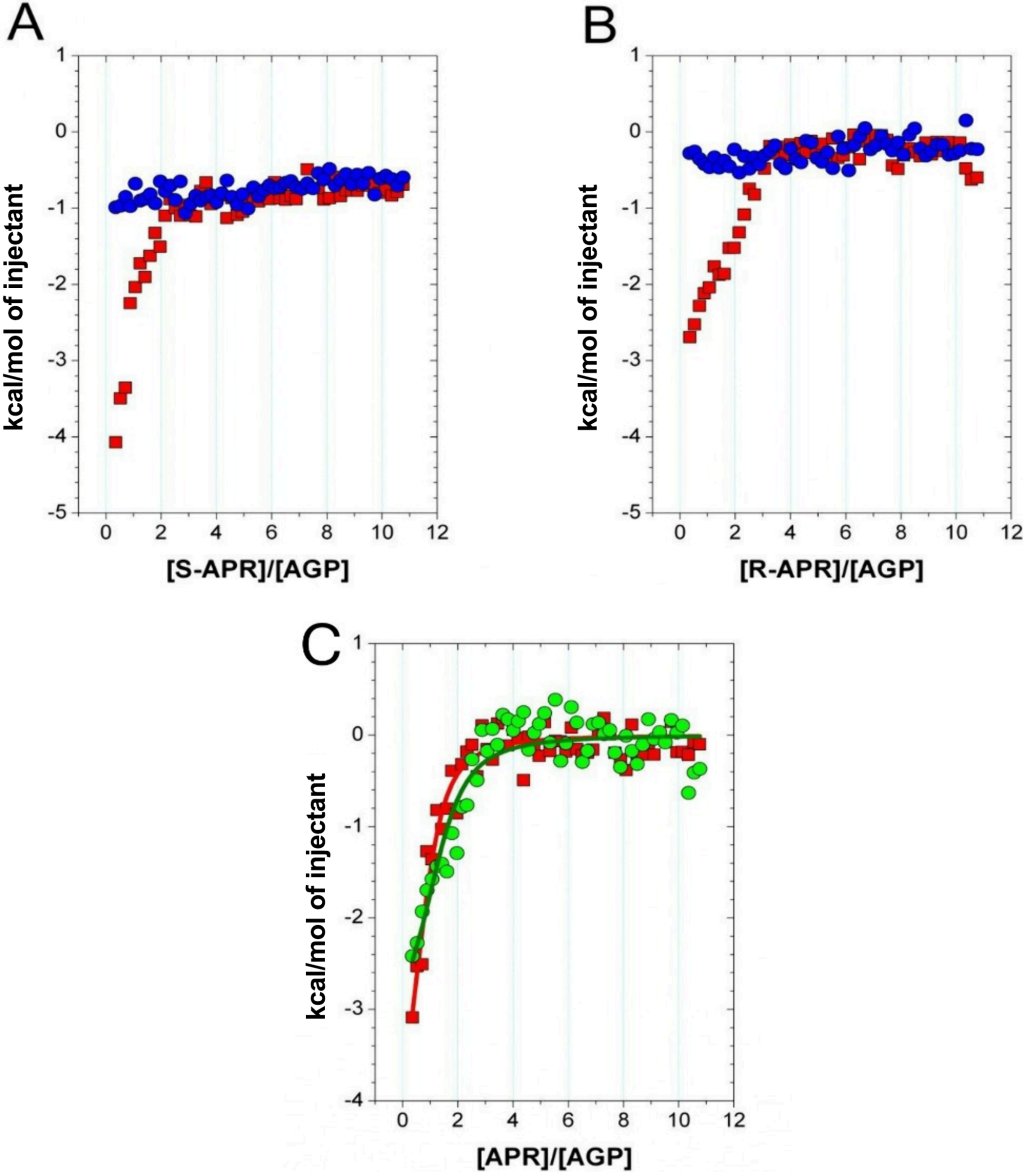
Thermal effects of 10 μM AGP protein solution titrated with 500 μM APR solution (■) as a function of molar ratio of APR to AGP and the corresponding thermal effects of APR dilution (●) in aqueous 5% DMSO at 25 °C for (A) S-APR and (B) R-APR. (C) Thermal effects of the direct interaction between AGP protein and APR in aqueous 5% DMSO at 25 °C, described with the One Set of Sites model (solid line) for S-APR (■) and R-APR (●).

**Table 3:** Stoichiometric Parameter (n), Logarithm of Binding Constant (log K), and Standard Enthalpy (ΔH), Entropy (ΔS), and Gibbs Free Energy (ΔG) of APR Binding with AGP Protein in Aqueous 5% DMSO at 25 °C

Ligand	n	log K	ΔH (kcal mol^–1^)	ΔS (cal K^–1^ mol^–1^)	ΔG (kcal mol^–1^)
S-APR	0.8 ± 0.2	5.55 ± 0.09	–4.80 ± 0.27	9.3 ± 1.3	–7.57 ± 0.12
R-APR	1.3 ± 0.3	5.48 ± 0.14	–3.40 ± 0.26	13.7 ± 1.5	–7.48 ± 0.18

The obtained stoichiometry, binding constants, and standard thermodynamic parameters (Table 3) indicate that AGP possesses an active site capable of binding S-APR or R-APR in a spontaneous (ΔG < 0) and exothermic (ΔH < 0) manner. These results suggest that the direct interaction between the APR enantiomers and AGP predominates over the energetic contributions from partial desolvation of the ligand and the binding site. The positive entropy change (ΔS > 0) observed during AGP binding with both APR enantiomers suggests an increase in system disorder, likely resulting from the release of ordered solvent molecules upon complex formation.^36,37^ Binding constants (log K > 5) of AGP with both S-APR and R-APR are similar and indicate that both enantiomers form stable supramolecular complexes with the protein. Interactions of AGP with S-APR are more exothermic than those with R-APR, which suggests that the protein interacts more strongly with S-APR than with R-APR. Binding of R-APR to AGP is associated with a slightly greater increase in the disorder of the system compared to that of S-APR.

#### Ultracentrifugation Experiments

In a concentration-dependent fashion, AGP gradually decreased the levels of both S-APR and R-APR in the supernatants (Figure 4), suggesting the formation of stable APR–AGP complexes. The protein induced smaller changes in R-APR versus S-APR levels, demonstrating that these isomers bind to AGP with similar stability; however, the stability of the S-APR–AGP complex is slightly higher. It is also in accordance with the binding constants determined based on eq 3 for the S-APR–AGP (log K = 5.77 ± 0.17) and R-APR–AGP (log K = 5.64 ± 0.15) complexes, respectively (Figure 4).

**Figure 4:**
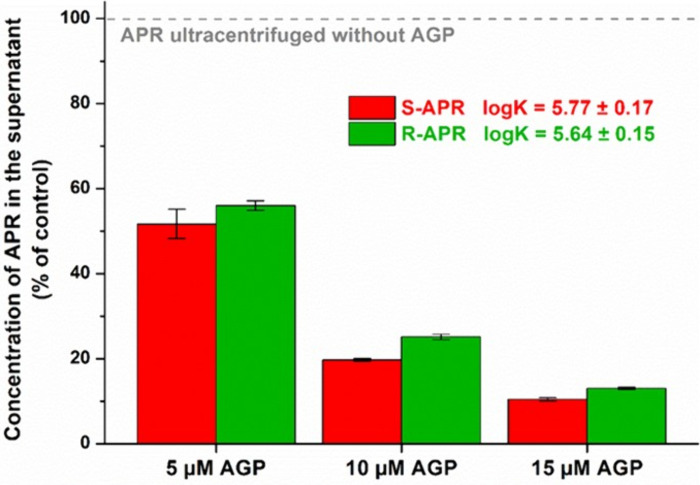
Concentration (% of control) of S-APR or R-APR (both 5 μM) in the supernatant after ultracentrifugation in the presence of increasing levels of AGP (5, 10, or 15 μM) in PBS (pH 7.4). Means ± SDs are represented (n = 3), where 100% marks the concentration of APR in the supernatant after ultracentrifugation without the protein.

### Molecular Docking

Molecular docking calculations were conducted to determine the interactions between APR enantiomers and human AGP variants (Figure 5). The complexes with the strongest interactions were selected and analyzed. In the case of variant A, five direct interactions were identified between the residues of the protein and S-APR, and four of those are hydrogen bonding (Table 4, left; Figure 5, top). In contrast, in the case of R-APR–AGP (A variant), only three direct interactions were established; although all of them were hydrogen bonding, two of them were created with the AGP residues TYR37 and GLU92, which were also involved in interactions with S-APR (Table 4, left; Figure 5, top). The third interaction between R-APR and AGP was established between SER127 and one of the oxygen atoms of the methyl sulfonyl group of the ligand. This pattern occurs in the other complex as well, but there, the interacting residue is TYR37. Regarding the S-APR–AGP complexes, TYR37 established an interaction with the carbonyl of the isoindole unit of the ligand. Overall, if we consider the number of interactions, S-APR established one extra hydrogen bond and an extra π–π interaction with the A variant of AGP compared to R-APR. In the case of the F1S variant, the number of interactions is the same; three direct interactions were established in both S- and R-APR complexes, but the nature of the interactions is different. S-APR established a π–cation interaction with ARG90 and two hydrogen bonds with SER125 and GLN66, while R-APR established one hydrogen bond with GLU64 and two π–π interactions with TYR27 and PHE114 (Table 4, right).

**Table 4:** Interactions between S-APR and R-APR and Two Human AGP Variants^a^

Interacting AGP residues: A variant	S-APR	R-APR	Interacting AGP residues: F1S variant	S-APR	R-APR
TYR37	YES	YES			
GLN66	YES	NO	GLN66	YES	NO
ARG90	YES	NO			
GLU92	YES	YES			
PHE112	YES	NO			
SER125	NO	YES	SER125	YES	NO
			PHE114	NO	YES
			TYR27	NO	YES
			GLU64	NO	YES
			ARG90	YES	NO

a YES, interaction established; NO, no interaction observed.

**Figure 5:**
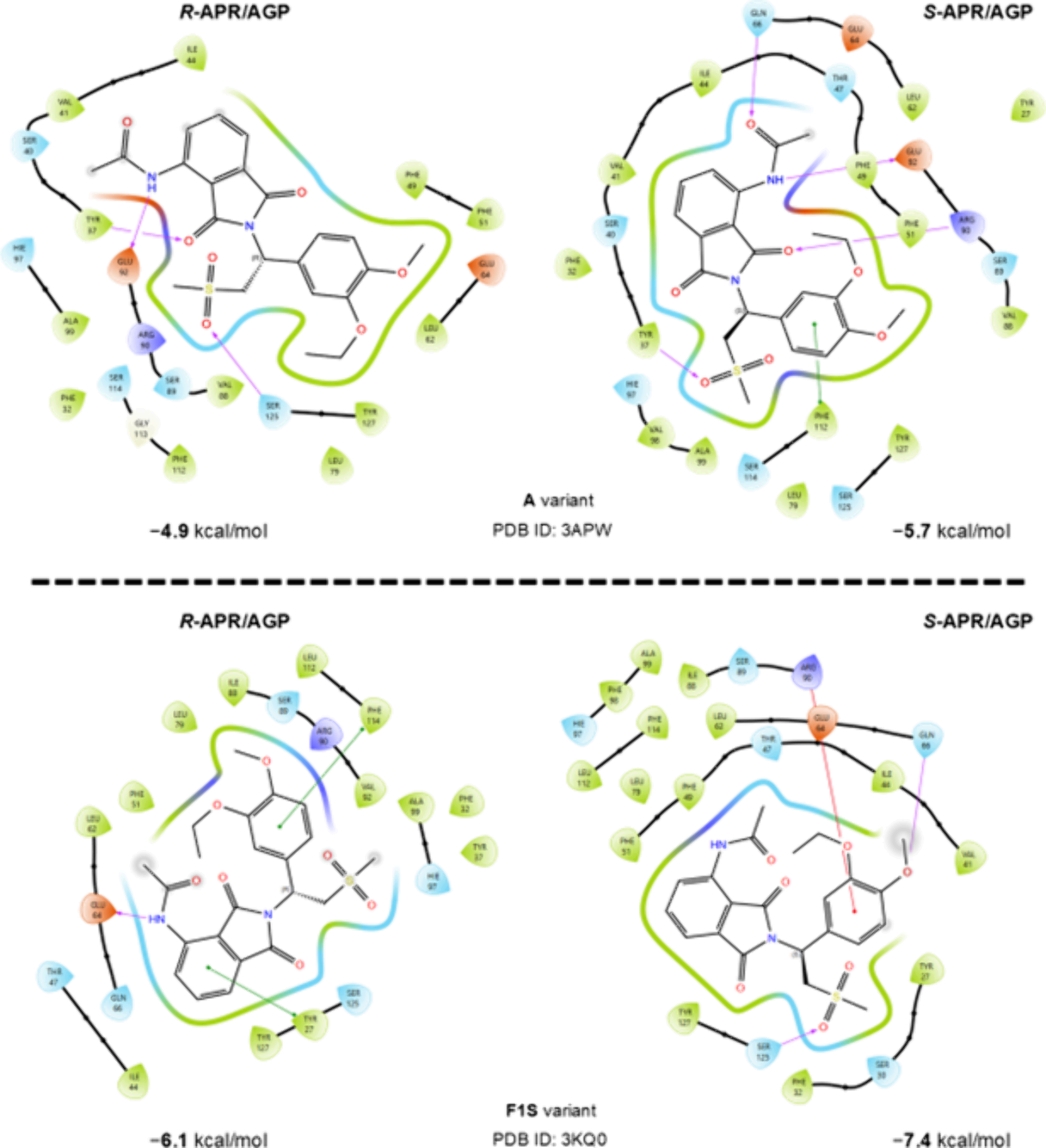
Ligand interaction maps and the corresponding docking scores of the R-APR–AGP and S-APR–AGP complexes. Both the A and F1S variants of AGP were considered.

In both A and F1S variants, more direct or stronger interactions led to stronger complexation, as the docking score for R-APR is only −4.9 and −6.1 kcal mol^–1^, respectively, while it is −5.7 and −7.4 kcal mol^–1^ for S-APR, respectively (Figure 5). It is noteworthy that the molecular docking results corroborated the experimental findings, indicating preferential binding of the S-enantiomer of APR over the R-enantiomer to human AGP.

### Stereoselective Binding of Apremilast to Different Plasma Proteins

In the public assessment report of Otezla, it is stated that the original drug contains S-APR as the active ingredient; however, the transport protein binding of APR was not investigated.^38^ Based on this document, we know only that overall plasma protein binding is highly species-specific: across the tested concentration range of 0.25 to 2.5 μg/mL, the average percentage of APR bound to plasma proteins was 88.6% in mouse plasma, 90.6% in rat plasma, 80.9% in rabbit plasma, 84.3% in monkey plasma, and 68.3% in human plasma. There is a more than 20% difference in plasma binding between rat and human and a 16% difference between monkey and human. Enantioselective pharmacokinetic parameters were also not investigated. Based on our results regarding APR plasma protein binding, APR binds to the main transport proteins in a stereoselective manner. Table 5 presents the stability constants (log K values) for both enantiomers with AGP and HSA. The difference between the log K values of the individual enantiomers is greater for HSA, indicating that enantioselective binding is more pronounced in the case of HSA. However, the binding stability values for AGP are much higher than those for HSA, suggesting that AGP plays a crucial role in the plasma transport of APR. It is known that the concentration of HSA in plasma is approximately 50 times higher than that of AGP under normal physiological conditions. Nevertheless, AGP levels increase in inflammatory diseases such as psoriasis and psoriatic arthritis, further reinforcing the role of AGP in the plasma protein binding of APR.

**Table 5:** Stability Constants (log K) for S-APR and R-APR Binding to AGP and HSA

Enantiomer	AGP	HSA^a^
S-APR	5.77 ± 0.17	4.02 ± 0.08
R-APR	5.64 ± 0.15	3.81 ± 0.05

a From ref 33.

## Conclusion

Our study provides a comprehensive characterization of the enantioselective binding of APR enantiomers to AGP using multiple complementary techniques. The results consistently demonstrated that both enantiomers form stable complexes with AGP, but the S-enantiomer exhibits a slight preference in binding strength, as revealed by HPLC, ITC, ultracentrifugation, and molecular docking. The stronger and more specific interaction between S-APR and AGP is due to the formation of additional hydrogen bonds and π–π interactions. These findings enhance our understanding of the enantioselective protein binding of APR, which may have implications for its pharmacokinetics and efficacy in clinical settings. The study highlights the importance of using a multitechnique strategy to unravel the complex nature of drug–protein interactions, particularly for chiral pharmaceuticals.

## Materials and Methods

### Materials

APR enantiomers were obtained from Beijing Mesochem Technology Co., Ltd. (Beijing, China). α_1_-Acid glycoprotein (AGP, >99% purity by agarose gel electrophoresis) was purchased from Sigma-Aldrich (St. Louis, MO, USA). HPLC-grade 2-propanol (IPA) was supplied by Merck (Darmstadt, Germany). The chiral AGP column (ChromTech Chiral-AGP) with dimensions of 150 mm × 4.0 mm was sourced from ChromTech Ltd. (Congleton, UK). Analytical-grade reagents and solvents, including dimethyl sulfoxide (DMSO), acetic acid, phosphoric acid, sodium phosphate, sodium hydroxide, ammonium bicarbonate, and ammonium acetate, were obtained from Sigma-Aldrich (Budapest, Hungary). All chemicals and solvents were used as received, without further purification. Deionized water was prepared using a Milli-Q Direct 8 purification system (Millipore Corporation, Bedford, MA, USA).

### HPLC System for Investigating Stereoselective Binding

LC-UV analysis was conducted on an Agilent 1100 HPLC system that included a quaternary pump, an autosampler, a column compartment, and a DAD detector (Agilent Technologies, Waldbronn, Germany). Data analysis was performed with ChemStation software (version: B04.03-SP2). UV detection was set at 230 nm. Stock solutions were prepared in methanol at a concentration of 1 mg/mL (2.17 mM), and further dilutions were made by using deionized water. Samples were prepared to contain 0.1 mg/mL (0.217 mM) R-APR and 0.2 mg/mL (0.434 mM) S-APR, with each analysis performed using a 5 μL injection volume. Sodium phosphate, ammonium carbonate, and ammonium acetate buffers were prepared at a concentration of 10 mM and titrated with 0.1 M sodium hydroxide, 0.1 M phosphoric acid, 0.1 M ammonia solution, or 0.1 M acetic acid to a pH of 7.0. Chromatographic data were evaluated according to standard calculation methods. The retention factor (k) for each enantiomer was determined using the following equation:

$$k=\left(t_{r}-t_{0}\right)/t_{0}$$ 1

where t_r_ denotes the retention time of the target enantiomer and t_0_ represents the column dead time.

Additionally, the retention factor was used to estimate the percentage of protein-bound analytes according to the following formula:

b%=100×k/(1+k) 2

Three parallel measurements were performed for each case.

### Isothermal Titration Calorimetry

ITC was performed using a VP-ITC calorimeter (MicroCal) to measure the heat effects of titrating 10 μM AGP solutions with 500 μM S-APR or R-APR in aqueous 5% (v/v) DMSO (pH 6.9) at 25 °C. The working cell contained 1427.5 μL of AGP solution, which was titrated with 5 μL aliquots of the APR solution from the syringe, up to a total volume of 275 μL. The reference cell was filled with aqueous 5% DMSO. Each injection lasted 10 s, with a 1000 s interval between injections. The stirring speed was maintained at 307 rpm. Control experiments were also conducted to measure the heat of dilution by injecting S-APR or R-APR solutions into the solvent under identical conditions.

The heat effects resulting from the direct interaction of the AGP protein with the ligands (S-APR or R-APR) were determined by subtracting the heat effects of ligand dilution from those obtained during the titration of AGP with the corresponding ligand. The resulting isotherm was next fitted with the nonlinear multiparameter regression method (Origin 7.0 MicroCal) using the One Set of Sites model,^39^ which assumes that a receptor (macromolecule) has a single class of multiple independent binding sites for a ligand. In this model, each site has the same affinity and thermodynamics for the ligand, and the binding of one ligand molecule to a site is independent of the binding events at other sites. Using this model to analyze ITC results, one can calculate the following binding parameters: stoichiometry n of the complex, describing the number of binding sites in the macromolecule (here protein), and binding (association) constant K (L mol^–1^) as well as standard thermodynamic functions (enthalpy, entropy, and Gibbs free energy) of the ligand (here S-APR or R-APR) binding with the active site of the macromolecule by fitting the heat effects of direct interactions with the equation^39^

$$q=\frac{{\mathrm{nM}}_{t}\Delta {\mathrm{HV}}_{0}}{2}\left[1+\frac{X_{t}}{{\mathrm{nM}}_{t}}+\frac{1}{{\mathrm{nKM}}_{t}}-\sqrt{{\left(1+\frac{X_{t}}{{\mathrm{nM}}_{t}}+\frac{1}{{\mathrm{nKM}}_{t}}\right)}^{2}-\frac{4X_{t}}{{\mathrm{nM}}_{t}}}\right]$$

where M_t_ and X_t_ are the concentrations of the macromolecule (protein) and its ligand (S-APR or R-APR), V_0_ is the working volume of the cell (here 1.43 mL), n is stoichiometric parameter describing the number of ligand molecules bound by the macromolecule, K is the equilibrium constant of the ligand binding with the active site of the macromolecule, and ΔH is the standard molar enthalpy of the ligand binding with the active site of the macromolecule. The standard Gibbs free energy of binding ΔG and standard entropy of binding ΔS can be next calculated using the basic thermodynamic relationship

ΔG=ΔH−TΔS=−RTln⁡K

where T is temperature and R is the gas constant.

### Ultracentrifugation Experiments

The interaction of S-APR and R-APR with AGP was also examined with ultracentrifugation, where increasing protein levels (final concentrations: 0, 5, 10, and 15 μM) were added to a standard amount of the ligand (5 μM) in PBS (pH 7.4). These samples (600 μL) were centrifuged for 16 h at 170,000g and 20 °C employing an Optima MAX-XP ultracentrifuge (Beckman Coulter, Brea, CA, USA). Using these experimental conditions, the protein is slowly sedimented without the disruption of ligand–protein interactions.^20–23,40^ Thereafter, the unbound free fraction of APR was directly quantified in the protein-free supernatants by the HPLC-UV method (see the next paragraph). Binding (association) constants (K; unit: L mol^–1^) of APR–AGP complexes were calculated assuming a 1:1 stoichiometry of complex formation:^21,23^

K=([APR−AGP])/([APR]×[AGP]) 3

where [APR] is the molar concentration of the unbound free ligand, [AGP] is the molar concentration of the unbound free protein, and [APR–AGP] indicates the molar concentration of the ligand–protein complex.

The APR concentration after centrifugation was measured by a validated HPLC-UV method using an XTERRA RP18 column (5 μm, 4.6 × 100 mm), maintained at 40 °C. Isocratic elution was applied using acetonitrile and water containing 0.5% formic acid (60:40, v/v) as the mobile phase. The flow rate was set to 1.0 mL/min. The injection volume was 20 μL, and detection was performed at 344 nm. The concentration was determined by using the external standard method. A stock solution of APR (5 mg/mL, 10.86 mM) was prepared in DMSO and further diluted with PBS to obtain the working standards. The calibration curve was constructed using standard solutions at the following concentrations: 0.5, 1.5, 2.5, 3.0, 4.5, 6.0, and 7.5 μM. The method demonstrated excellent linearity across the tested concentration range, with correlation coefficients (r^2^) exceeding 0.9993.

### Docking Study

During molecular docking, two variants of human AGP were employed as the host, and the corresponding structures were downloaded from the RCSB Protein Data Bank (RCSB PDB) (PDB IDs: 3APW and 3KQ0). To prepare the host structure, Protein Preparation Wizard^41^ was used, and nonprotein species were deleted by employing default settings. The protonation states of AGP residues at physiological pH were predicted using the PROPKA algorithm.^42^ Additionally, system optimization was performed using the OPLS3e force field parameters.^43^ The binding site of the native ligand in the PDB structure was used to define the target region for docking calculations. The APR enantiomers were prepared as guest molecules using LigPrep with the default settings. The receptor grid for docking calculations was generated using the Receptor Grid Generation module, and molecular docking was subsequently performed with Glide.^44–46^ During the docking, a flexible process was considered, and extra precision (XP) mode was selected. The resulting docking scores were compared and analyzed. All calculations were conducted by using the Schrödinger suite (Release 2020-4).
